# Synthesis, crystal structure and Hirshfeld surface analysis of 3,3′-[ethane-1,2-diylbis(sulfanedi­yl)]bis­(1*H*-1,2,4-triazol-5-amine)

**DOI:** 10.1107/S2056989026003464

**Published:** 2026-04-10

**Authors:** Shakhnoza Mavlonova, Giyosiddin Khayrullaev, Dilnoza Rakhmonova, Mirigul Berdimuratova, Shakhnoza Kadirova, Jamshid Ashurov, Batirbay Torambetov

**Affiliations:** a National University of Uzbekistan named after Mirzo Ulugbek 4 University St Tashkent 100174 Uzbekistan; b Karakalpak State University, 1 Ch. Abdirov St. Nukus, 230112, Uzbekistan; c Institute of Bioorganic Chemistry, Academy of Sciences of Uzbekistan, M. Ulugbek St., 83, Tashkent, 100125, Uzbekistan; University of Neuchâtel, Switzerland

**Keywords:** mol­ecular structure, crystal structure, 1,2,4-triazole, hydrogen bond, Hirshfeld analysis

## Abstract

The title compound, C_6_H_10_N_8_S_2_, crystallizes in the monoclinic crystal system with *P*2_1_/*c* space group. The mol­ecular geometry features a flexible ethyl­enedi­thio spacer inducing a 76.69 (11)° dihedral angle between triazole moieties; this twist precludes π–π stacking. The crystal cohesion is instead driven by a two-dimensional supra­molecular framework maintained by strong N—H⋯N hydrogen bonds and auxiliary N⋯S and C⋯S contacts. Qu­anti­tative Hirshfeld surface analysis confirms the dominance of hydrogen-involving inter­actions (98.8%), with N⋯H (40.4%) and H⋯H (27.1%) as the primary contributors to the packing arrangement.

## Abstract 2

The mol­ecular and crystal structure of the 3,3′-[ethane-1,2-diylbis(sulfanedi­yl)]bis­(1*H*-1,2,4-triazol-5-amine) were studied and Hirshfeld surfaces and fingerprint plots were generated to investigate the various inter­molecular inter­actions.

## 1. Chemical context

The 1,2,4-triazole ring is an important five-membered heterocyclic scaffold containing three nitro­gen atoms that impart distinctive electronic characteristics and a relatively high dipole moment (Kaur & Chawla, 2017 ▸; El–Sebaey, 2020 ▸; Naeem, *et al.*, 2025 ▸). It has attracted considerable inter­est in coordination chemistry, where the differing nucleophilicity enables diverse coordination modes, including monodentate, bidentate, and bridging arrangements (Zhang *et al.*, 2008 ▸; Deswal *et al.*, 2024 ▸; Bodurlar *et al.*, 2025 ▸; Bader *et al.*, 2020 ▸). Derivatives of 1,2,4-triazole are also well known for their wide range of biological activities, such as anti­cancer, anti­oxidant, analgesic, anti­malarial, anti­tuberculosis, insecticidal, anti­mycobacterial, anti­microbial, anti­convulsant, anti-inflammatory, anti­fungal, and anti­bacterial properties (El-Sherief *et al.*, 2018 ▸; Sathyanarayana & Poojary, 2020 ▸; Wen *et al.*, 2020 ▸; Gultekin *et al.*, 2018 ▸). Representative examples of triazole are reported by Nuralieva *et al.* (2025 ▸), Pirimova *et al.* (2022 ▸), and Torambetov *et al.* (2025 ▸). Such compounds function as multitopic ligands bearing both thiol (–SH) and amine (–NH_2_) functional groups. The presence of this soft sulfur and hard nitro­gen donor atoms allows these mol­ecules to participate in a range of coordination environments, facilitating the formation of complex, high-dimensional crystalline architectures stabilized by extensive hydrogen-bonding networks in metal complexes (Lin *et al.*, 2017 ▸; Ma *et al.*, 2008 ▸; Rakova *et al.*, 2003 ▸). As a continuation of our previous work (Khayrullaev, *et al.*, 2023 ▸), we report here the synthesis and single-crystal structural characterization of 3,3′-[ethane-1,2-diylbis(sulfane­di­yl)]bis­(1*H*-1,2,4-triazol-5-amine), a derivative containing two (3-amino-1,2,4-triazol-5-yl)sulfanyl units inter­connected through an ethyl­ene spacer.
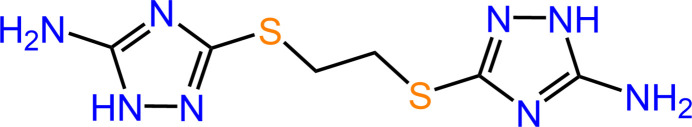


## 2. Structural commentary

**Figure 1:**
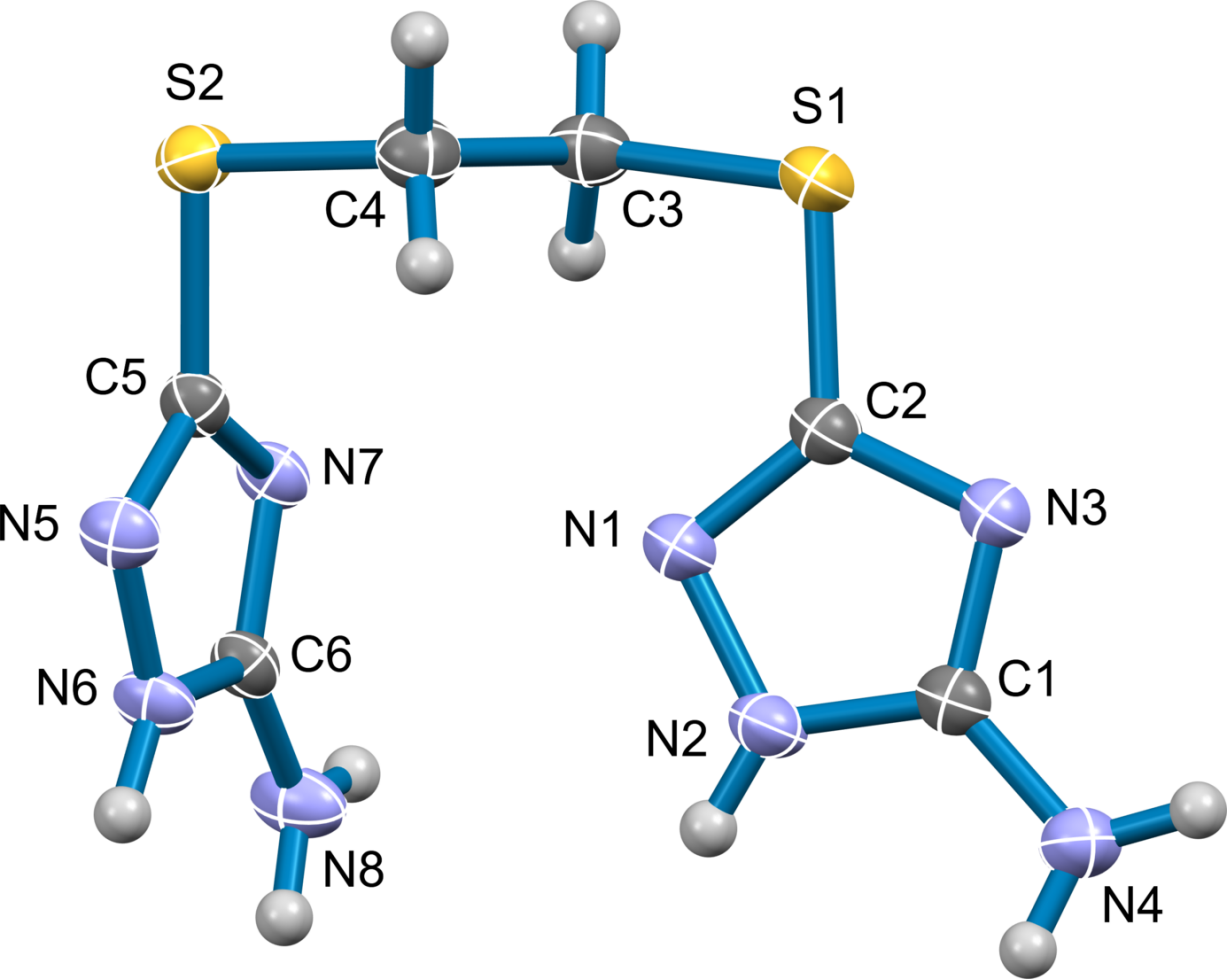
The title compound with displacement ellipsoids at the 50% probability level. For visual clarity, hydrogen atoms are represented as spheres of arbitrary size.

The title compound crystallizes in the monoclinic system in the *P*2_1_/c (No. 14) space group with one mol­ecule in the asymmetric unit (Fig. 1 ▸). The mol­ecule consists of two (3-amino-1,2,4-triazol-5-yl)sulfanyl units bridged by an ethyl­ene spacer. The mol­ecular geometry is characterized by a pronounced non-coplanar orientation, with the two triazole moieties separated by a dihedral angle of 76.69 (11)° between their mean planes. This significant twist in the mol­ecular backbone is attributed to the conformational flexibility of the ethyl­enedi­thio spacer. Consequently, this nearly orthogonal orientation prevents the triazole rings from achieving the facial alignment necessary for π–π stacking, shifting the burden of crystal consolidation onto the extensive hydrogen-bonding network.

## 3. Supra­molecular features

**Figure 2:**
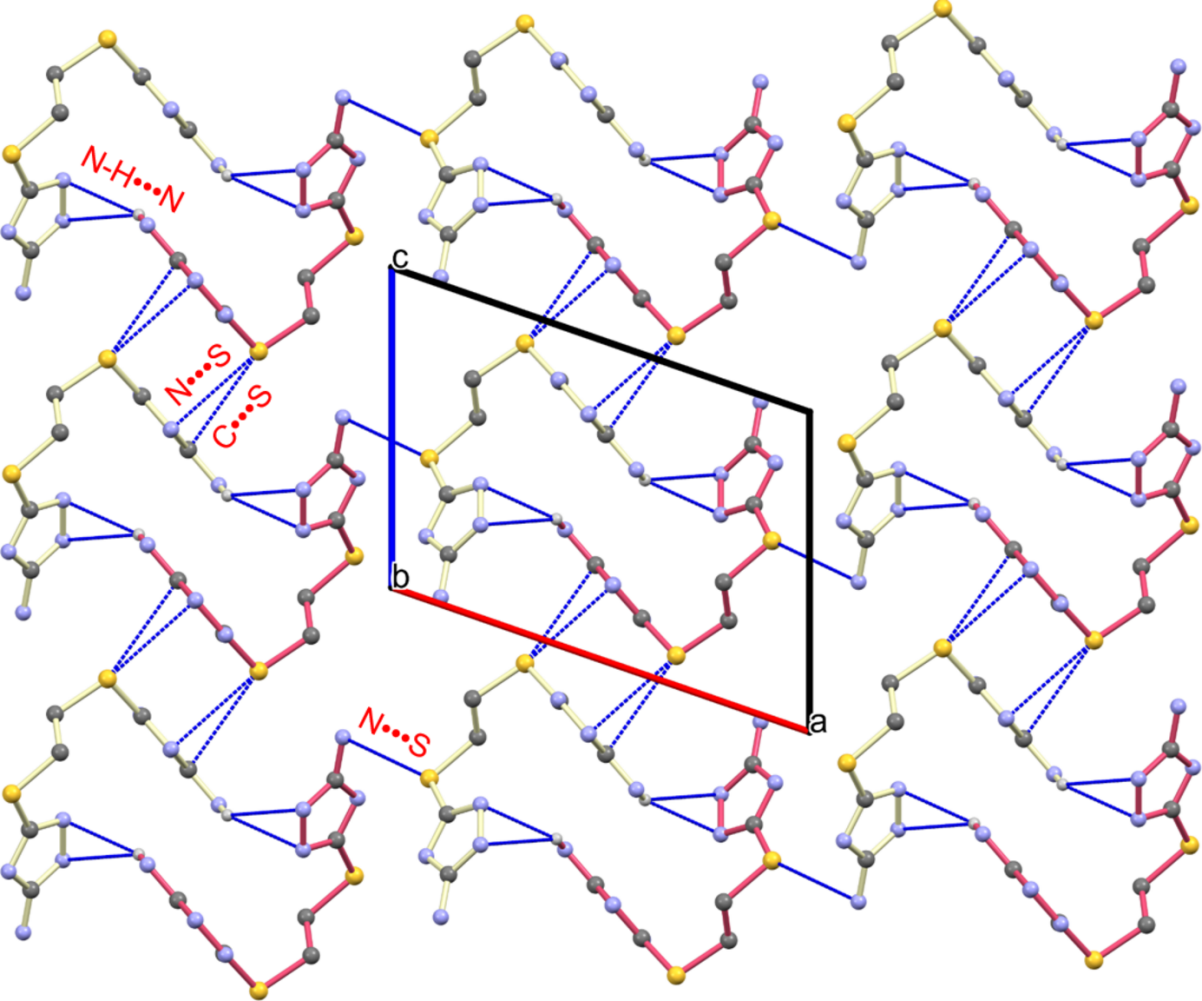
Packing arrangement viewed along the *b* axis, illustrating the network of inter­molecular N—H⋯N, N⋯S, and C⋯S inter­actions.

The crystal packing is governed by a sophisticated network of non-covalent inter­actions rather than traditional stacking motifs. A view of the packing diagram along the *b*-axis reveals that adjacent 1D mol­ecular chains are linked via N—H⋯N [N8–H8*A*⋯N1 = 2.62 (3) Å; Table 1 ▸] hydrogen bonds, which facilitate the assembly of mol­ecular units along the *a*-axis direction (Fig. 2 ▸). Beyond the primary hydrogen-bonding inter­actions, the structural architecture is further reinforced by auxiliary N⋯S [3.361 (2) Å] and C⋯S [3.525 (2) Å] inter­molecular contacts. Although these inter­actions are weaker than N—H⋯N inter­actions, the heteroatom contacts collectively bridge the mol­ecular layers, consolidating the 2D supra­molecular framework. The 76.69 (11)° dihedral twist previously mentioned precludes any π–π stacking, thereby increasing reliance on these specific hydrogen bonds and sulfur-mediated contacts for overall crystal cohesion.

## 4. Hirshfeld surface and fingerprint analysis

**Figure 3:**
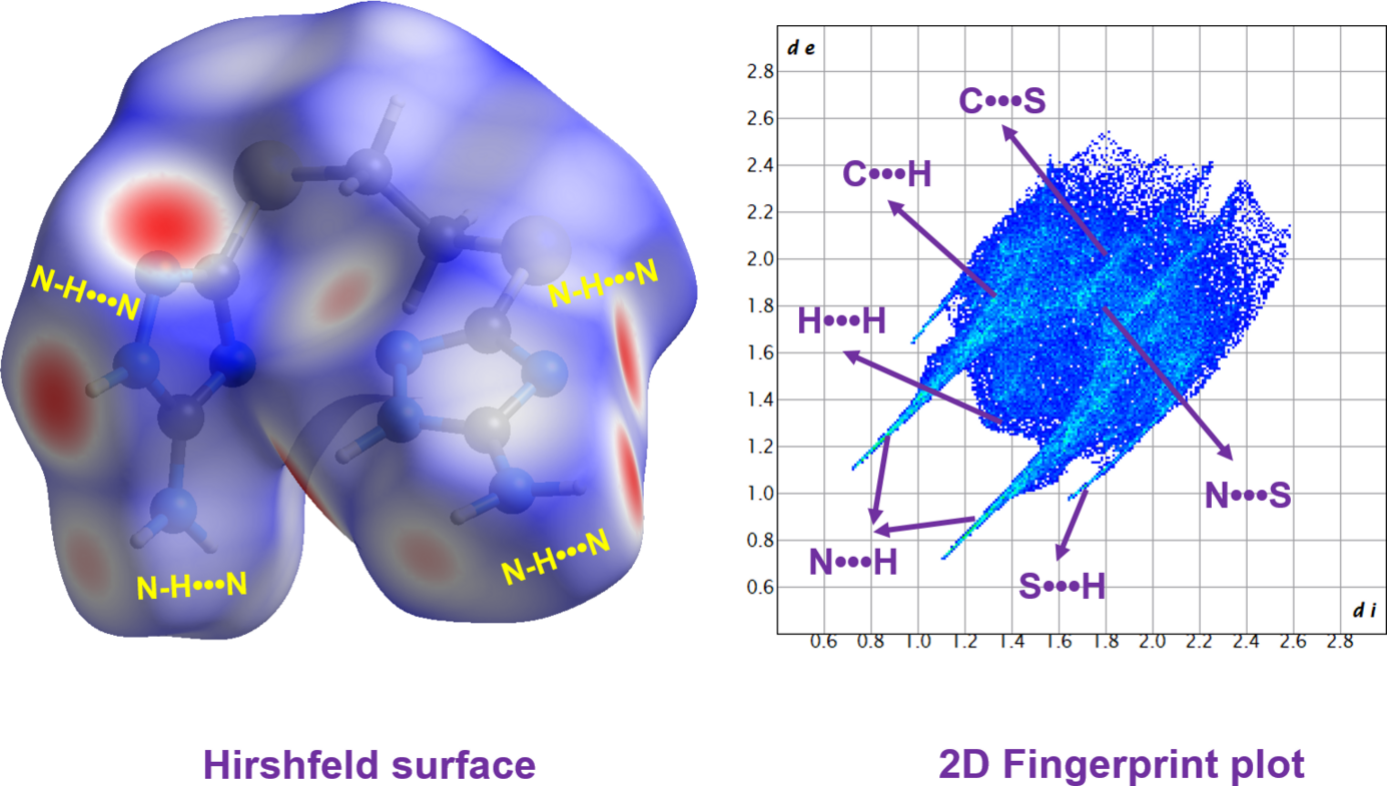
Hirshfeld surface and two-dimensional fingerprint plot.

Hirshfeld surface (HS) analysis (Spackman & Jayatilaka, 2009 ▸) and two-dimensional fingerprint plots (Spackman & McKinnon, 2002 ▸) were performed using *CrystalExplorer* (Spackman *et al.*, 2021 ▸). Qu­anti­tative Hirshfeld surface analysis demonstrates that the crystal packing is primarily consolidated by hydrogen-involving inter­actions, which account for a substantial 98.8% of the total surface area. The inter­molecular contact distribution is dominated by N⋯H (40.4%), followed by H⋯H (27.1%), S⋯H (17.9%), C⋯H (5.1%), and minor contributions from C⋯S (4.1%) and N⋯S (4.1%). The Hirshfeld surface displays prominent dark-red spots, signifying close contacts that are significantly shorter than the sum of the van der Waals radii (Fig. 3 ▸). These spots are primarily attributed to strong N—H⋯N hydrogen bonding between adjacent mol­ecular units. This is further corroborated by the 2D fingerprint plots, which reveal characteristic spikes for N⋯H inter­actions at approximately *d*_i_ + *d*_e_ = 1.8 Å and H⋯H contacts at *d*_i_ + *d*_e_ = 2.6 Å.

## 5. Database survey

A search of the Cambridge Structural Database (CSD) using the ConQuest program (Version 6.01, November 2025; Groom *et al.*, 2016 ▸) identified only 44 crystal structures containing the 3-amino-5-mercapto-1,2,4-triazole moiety. Of these, 25 are organic compounds and 19 are metal-based systems incorporating Fe, Co, Ni, Cu, Ag, Cd, Sn, Pr, Ho, Er, and Re. Among these structures, only two compounds contain two triazole moieties within the same mol­ecule, in which the triazole units are linked by a di­sulfide bridge (DILZIL, Khayrullaev *et al.*, 2023 ▸; SEDMEV, Yang *et al.*, 2012 ▸). Notably, to date, no crystal structure has been reported featuring two (3-amino-1,2,4-triazol-5-yl)sulfanyl moieties linked by an ethyl­ene spacer, underscoring the novelty of the present study.

## 6. Synthesis and crystallization

3-Amino-5-mercapto-1,2,4-triazole (1.16 g, 0.01 mol) and KOH (0.56 g, 0.01 mol) were dissolved in methanol (25 mL). The reaction mixture was cooled to 273 K, and 1,2-di­chloro­ethane (0.005 mol) was added dropwise with stirring. The mixture was then refluxed at 338 K for 8 h. The reaction progress was monitored by thin-layer chromatography (TLC). After completion of the reaction, the solvent was removed under reduced pressure. The residue was dissolved in water (30 mL) and extracted with ethyl acetate (3 × 30 mL). The combined organic layers were dried over anhydrous Na_2_SO_4_, filtered, and concentrated under vacuum. The resulting solid was dried at room temperature for 4 days to afford colourless crystals (85% yield). The crude crystals was recrystallized from methanol solution.

## 7. Refinement

Crystal data, data collection and structure refinement details are summarized in Table 2 ▸. All hydrogen atoms were located from difference-Fourier maps and refined isotropically.

## Supplementary Material

Crystal structure: contains datablock(s) I. DOI: 10.1107/S2056989026003464/tx2108sup1.cif

Structure factors: contains datablock(s) I. DOI: 10.1107/S2056989026003464/tx2108Isup3.hkl

Supporting information file. DOI: 10.1107/S2056989026003464/tx2108Isup3.cml

CCDC reference: 2543358

Additional supporting information:  crystallographic information; 3D view; checkCIF report

## Figures and Tables

**Table 1:** Hydrogen-bond geometry (Å, °)

*D*—H⋯*A*	*D*—H	H⋯*A*	*D*⋯*A*	*D*—H⋯*A*
N6—H6⋯N7^i^	0.86	1.99	2.838 (2)	167
N2—H2⋯N5^ii^	0.86	2.05	2.877 (2)	160
N8—H8*A*⋯N1^iii^	0.76 (3)	2.62 (3)	3.232 (3)	140 (2)
N4—H4*A*⋯N1^ii^	0.83 (3)	2.56 (3)	3.346 (3)	159 (2)
N8—H8*B*⋯S2^i^	0.85 (3)	2.80 (3)	3.615 (2)	162 (3)
N4—H4*B*⋯N3^iv^	0.90 (3)	2.13 (3)	3.010 (3)	168 (3)

Symmetry codes: (i) 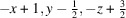; (ii) 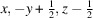; (iii) 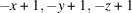; (iv) 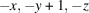.

**Table 2:** Experimental details

Crystal data	
Chemical formula	C_6_H_10_N_8_S_2_
*M*_r_	258.34
Crystal system, space group	Monoclinic, *P*2_1_/*c*
Temperature (K)	293
*a*, *b*, *c* (Å)	12.7401 (2), 9.8361 (1), 9.2113 (2)
β (°)	109.069 (2)
*V* (Å^3^)	1090.95 (3)
*Z*	4
Radiation type	Cu *K*α
μ (mm^−1^)	4.35
Crystal size (mm)	0.18 × 0.12 × 0.1

Data collection	
Diffractometer	XtaLAB Synergy, Single source at home/near, HyPix3000
Absorption correction	Multi-scan (*CrysAlis PRO*; Rigaku OD, 2021 ▸)
*T*_min_, *T*_max_	0.615, 1.000
No. of measured, independent and observed [*I* > 2σ(*I*)] reflections	10263, 2107, 1927
*R*_int_	0.031
(sin θ/λ)_max_ (Å^−1^)	0.614

Refinement	
*R*[*F*^2^ > 2σ(*F*^2^)], *wR*(*F*^2^), *S*	0.035, 0.100, 1.09
No. of reflections	2107
No. of parameters	161
H-atom treatment	H atoms treated by a mixture of independent and constrained refinement
Δρ_max_, Δρ_min_ (e Å^−3^)	0.40, −0.24

Computer programs: *CrysAlis PRO* (Rigaku OD, 2021 ▸), *SHELXT2014/5* (Sheldrick, 2015*a* ▸), *SHELXL2016/6* (Sheldrick, 2015*b* ▸) and *OLEX2* (Dolomanov *et al.*, 2009 ▸).

## supplementary crystallographic information

### 3,3'-[Ethane-1,2-diylbis(sulfanediyl)]bis(1H-1,2,4-triazol-5-amine) Crystal data

**Table:**

C_6_H_10_N_8_S_2_	*F*(000) = 536
*M**_r_* = 258.34	*D*_x_ = 1.573 Mg m^−^^3^
Monoclinic, *P*2_1_/*c*	Cu *K*α radiation, λ = 1.54184 Å
*a* = 12.7401 (2) Å	Cell parameters from 6441 reflections
*b* = 9.8361 (1) Å	θ = 3.7–70.8°
*c* = 9.2113 (2) Å	µ = 4.35 mm^−^^1^
β = 109.069 (2)°	*T* = 293 K
*V* = 1090.95 (3) Å^3^	Block, colourless
*Z* = 4	0.18 × 0.12 × 0.1 mm

### 3,3'-[Ethane-1,2-diylbis(sulfanediyl)]bis(1H-1,2,4-triazol-5-amine) Data collection

**Table:**

XtaLAB Synergy, Single source at home/near, HyPix3000 diffractometer	2107 independent reflections
Radiation source: micro-focus sealed X-ray tube, PhotonJet (Cu) X-ray Source	1927 reflections with *I* > 2σ(*I*)
Mirror monochromator	*R*_int_ = 0.031
Detector resolution: 10.0000 pixels mm^-1^	θ_max_ = 71.3°, θ_min_ = 3.7°
ω scans	*h* = −15→15
Absorption correction: multi-scan (CrysAlisPro; Rigaku OD, 2021)	*k* = −12→11
*T*_min_ = 0.615, *T*_max_ = 1.000	*l* = −11→11
10263 measured reflections	

### 3,3'-[Ethane-1,2-diylbis(sulfanediyl)]bis(1H-1,2,4-triazol-5-amine) Refinement

**Table:**

Refinement on *F*^2^	Primary atom site location: dual
Least-squares matrix: full	Hydrogen site location: mixed
*R*[*F*^2^ > 2σ(*F*^2^)] = 0.035	H atoms treated by a mixture of independent and constrained refinement
*wR*(*F*^2^) = 0.100	*w* = 1/[σ^2^(*F*_o_^2^) + (0.0551*P*)^2^ + 0.2828*P*] where *P* = (*F*_o_^2^ + 2*F*_c_^2^)/3
*S* = 1.09	(Δ/σ)_max_ = 0.001
2107 reflections	Δρ_max_ = 0.40 e Å^−^^3^
161 parameters	Δρ_min_ = −0.24 e Å^−^^3^
0 restraints	

### 3,3'-[Ethane-1,2-diylbis(sulfanediyl)]bis(1H-1,2,4-triazol-5-amine) Special details

**Table:**

Geometry. All esds (except the esd in the dihedral angle between two l.s. planes) are estimated using the full covariance matrix. The cell esds are taken into account individually in the estimation of esds in distances, angles and torsion angles; correlations between esds in cell parameters are only used when they are defined by crystal symmetry. An approximate (isotropic) treatment of cell esds is used for estimating esds involving l.s. planes.

### 3,3'-[Ethane-1,2-diylbis(sulfanediyl)]bis(1H-1,2,4-triazol-5-amine) Fractional atomic coordinates and isotropic or equivalent isotropic displacement parameters (Å^2^)

**Table:**

	*x*	*y*	*z*	*U*_iso_*/*U*_eq_	
S2	0.31895 (4)	0.55253 (5)	0.90594 (6)	0.04855 (17)	
S1	0.08980 (5)	0.65665 (5)	0.44295 (6)	0.05777 (19)	
N7	0.47164 (12)	0.50710 (14)	0.75544 (17)	0.0401 (3)	
N6	0.47018 (13)	0.28482 (15)	0.75254 (19)	0.0452 (4)	
H6	0.485616	0.202665	0.734751	0.054*	
N3	0.08227 (13)	0.50853 (16)	0.19660 (18)	0.0458 (4)	
N5	0.39835 (13)	0.32119 (15)	0.82907 (19)	0.0459 (4)	
N2	0.21243 (14)	0.35730 (16)	0.2911 (2)	0.0493 (4)	
H2	0.255533	0.289118	0.295113	0.059*	
N1	0.21245 (14)	0.43632 (17)	0.41680 (19)	0.0490 (4)	
N8	0.58527 (17)	0.3923 (2)	0.6323 (3)	0.0594 (5)	
N4	0.11720 (17)	0.3516 (2)	0.0219 (2)	0.0551 (4)	
C5	0.40203 (14)	0.45463 (17)	0.8264 (2)	0.0390 (4)	
C6	0.51240 (14)	0.39592 (17)	0.7099 (2)	0.0391 (4)	
C1	0.13561 (15)	0.40294 (18)	0.1635 (2)	0.0435 (4)	
C2	0.13288 (15)	0.52359 (19)	0.3502 (2)	0.0434 (4)	
C4	0.19338 (17)	0.5601 (2)	0.7390 (2)	0.0521 (5)	
H4C	0.178660	0.471254	0.691014	0.063*	
H4D	0.130775	0.584750	0.771580	0.063*	
C3	0.20554 (18)	0.6624 (2)	0.6248 (3)	0.0548 (5)	
H3A	0.274469	0.645397	0.604546	0.066*	
H3B	0.209829	0.752709	0.668612	0.066*	
H8A	0.606 (2)	0.459 (3)	0.610 (3)	0.056 (7)*	
H4A	0.147 (2)	0.276 (3)	0.022 (3)	0.061 (7)*	
H8B	0.597 (2)	0.315 (3)	0.601 (3)	0.079 (9)*	
H4B	0.058 (3)	0.384 (3)	−0.053 (3)	0.078 (9)*	

### 3,3'-[Ethane-1,2-diylbis(sulfanediyl)]bis(1H-1,2,4-triazol-5-amine) Atomic displacement parameters (Å^2^)

**Table:**

	*U*^11^	*U*^22^	*U*^33^	*U*^12^	*U*^13^	*U*^23^
S2	0.0470 (3)	0.0517 (3)	0.0468 (3)	0.00188 (19)	0.0152 (2)	−0.00829 (19)
S1	0.0635 (3)	0.0539 (3)	0.0529 (3)	0.0241 (2)	0.0149 (2)	0.0000 (2)
N7	0.0423 (8)	0.0276 (7)	0.0507 (9)	−0.0013 (6)	0.0155 (7)	0.0015 (6)
N6	0.0512 (9)	0.0270 (7)	0.0612 (10)	0.0001 (6)	0.0238 (8)	−0.0008 (6)
N3	0.0443 (8)	0.0444 (8)	0.0483 (9)	0.0058 (7)	0.0145 (7)	0.0008 (7)
N5	0.0483 (8)	0.0344 (8)	0.0583 (10)	−0.0048 (6)	0.0220 (7)	−0.0004 (7)
N2	0.0513 (9)	0.0413 (8)	0.0576 (10)	0.0106 (7)	0.0209 (8)	0.0000 (7)
N1	0.0502 (9)	0.0451 (9)	0.0506 (9)	0.0114 (7)	0.0151 (7)	0.0003 (7)
N8	0.0710 (12)	0.0387 (10)	0.0846 (14)	0.0037 (9)	0.0474 (11)	0.0068 (9)
N4	0.0613 (11)	0.0509 (11)	0.0556 (11)	0.0043 (9)	0.0224 (9)	−0.0057 (8)
C5	0.0393 (9)	0.0329 (8)	0.0424 (9)	−0.0023 (7)	0.0101 (7)	−0.0020 (7)
C6	0.0405 (9)	0.0305 (8)	0.0453 (9)	−0.0007 (6)	0.0126 (7)	0.0018 (7)
C1	0.0421 (9)	0.0388 (9)	0.0532 (10)	−0.0026 (7)	0.0204 (8)	0.0012 (8)
C2	0.0414 (9)	0.0409 (9)	0.0492 (10)	0.0042 (7)	0.0164 (8)	0.0025 (8)
C4	0.0458 (10)	0.0536 (11)	0.0582 (12)	0.0014 (9)	0.0188 (9)	−0.0047 (9)
C3	0.0612 (12)	0.0431 (10)	0.0623 (13)	0.0015 (9)	0.0230 (10)	−0.0051 (9)

### 3,3'-[Ethane-1,2-diylbis(sulfanediyl)]bis(1H-1,2,4-triazol-5-amine) Geometric parameters (Å, º)

**Table:**

S2—C5	1.7580 (18)	N2—C1	1.338 (3)
S2—C4	1.822 (2)	N1—C2	1.317 (2)
S1—C2	1.7463 (19)	N8—C6	1.345 (3)
S1—C3	1.836 (2)	N8—H8A	0.76 (3)
N7—C5	1.363 (2)	N8—H8B	0.85 (3)
N7—C6	1.335 (2)	N4—C1	1.346 (3)
N6—H6	0.8600	N4—H4A	0.83 (3)
N6—N5	1.372 (2)	N4—H4B	0.90 (3)
N6—C6	1.332 (2)	C4—H4C	0.9700
N3—C1	1.330 (2)	C4—H4D	0.9700
N3—C2	1.358 (2)	C4—C3	1.500 (3)
N5—C5	1.314 (2)	C3—H3A	0.9700
N2—H2	0.8600	C3—H3B	0.9700
N2—N1	1.394 (2)		
			
C5—S2—C4	98.81 (9)	N7—C6—N8	126.52 (17)
C2—S1—C3	100.49 (9)	N6—C6—N7	110.08 (16)
C6—N7—C5	102.77 (14)	N6—C6—N8	123.40 (17)
N5—N6—H6	125.1	N3—C1—N2	110.01 (17)
C6—N6—H6	125.1	N3—C1—N4	125.09 (19)
C6—N6—N5	109.79 (14)	N2—C1—N4	124.88 (19)
C1—N3—C2	102.71 (15)	N3—C2—S1	118.24 (13)
C5—N5—N6	102.53 (14)	N1—C2—S1	125.31 (15)
N1—N2—H2	124.9	N1—C2—N3	116.44 (17)
C1—N2—H2	124.9	S2—C4—H4C	109.5
C1—N2—N1	110.14 (15)	S2—C4—H4D	109.5
C2—N1—N2	100.69 (16)	H4C—C4—H4D	108.1
C6—N8—H8A	119.0 (19)	C3—C4—S2	110.85 (15)
C6—N8—H8B	116 (2)	C3—C4—H4C	109.5
H8A—N8—H8B	124 (3)	C3—C4—H4D	109.5
C1—N4—H4A	113.2 (17)	S1—C3—H3A	109.1
C1—N4—H4B	116.8 (18)	S1—C3—H3B	109.1
H4A—N4—H4B	126 (2)	C4—C3—S1	112.54 (15)
N7—C5—S2	124.53 (13)	C4—C3—H3A	109.1
N5—C5—S2	120.63 (14)	C4—C3—H3B	109.1
N5—C5—N7	114.82 (16)	H3A—C3—H3B	107.8
			
S2—C4—C3—S1	171.73 (10)	C6—N7—C5—N5	0.7 (2)
N6—N5—C5—S2	177.82 (12)	C6—N6—N5—C5	0.3 (2)
N6—N5—C5—N7	−0.7 (2)	C1—N3—C2—S1	178.47 (14)
N5—N6—C6—N7	0.1 (2)	C1—N3—C2—N1	−0.6 (2)
N5—N6—C6—N8	−179.81 (18)	C1—N2—N1—C2	0.4 (2)
N2—N1—C2—S1	−178.83 (14)	C2—S1—C3—C4	−83.15 (16)
N2—N1—C2—N3	0.2 (2)	C2—N3—C1—N2	0.8 (2)
N1—N2—C1—N3	−0.7 (2)	C2—N3—C1—N4	−177.61 (18)
N1—N2—C1—N4	177.64 (18)	C4—S2—C5—N7	91.57 (16)
C5—S2—C4—C3	−77.89 (16)	C4—S2—C5—N5	−86.75 (16)
C5—N7—C6—N6	−0.48 (19)	C3—S1—C2—N3	−164.45 (15)
C5—N7—C6—N8	179.4 (2)	C3—S1—C2—N1	14.5 (2)
C6—N7—C5—S2	−177.68 (13)		

### 3,3'-[Ethane-1,2-diylbis(sulfanediyl)]bis(1H-1,2,4-triazol-5-amine) Hydrogen-bond geometry (Å, º)

**Table:**

*D*—H···*A*	*D*—H	H···*A*	*D*···*A*	*D*—H···*A*
N6—H6···N7^i^	0.86	1.99	2.838 (2)	167
N2—H2···N5^ii^	0.86	2.05	2.877 (2)	160
N8—H8*A*···N1^iii^	0.76 (3)	2.62 (3)	3.232 (3)	140 (2)
N4—H4*A*···N1^ii^	0.83 (3)	2.56 (3)	3.346 (3)	159 (2)
N8—H8*B*···S2^i^	0.85 (3)	2.80 (3)	3.615 (2)	162 (3)
N4—H4*B*···N3^iv^	0.90 (3)	2.13 (3)	3.010 (3)	168 (3)

Symmetry codes: (i) −*x*+1, *y*−1/2, −*z*+3/2; (ii) *x*, −*y*+1/2, *z*−1/2; (iii) −*x*+1, −*y*+1, −*z*+1; (iv) −*x*, −*y*+1, −*z*.

## References

[bb1] Bader, A. T., Rasheed, N. A., Aljeboree, M. & Alkaiml, A. F. (2020). *J. Phys. Conf. Ser.* **1664**, 012100.

[bb2] Bodurlar, Y., Ozturk, I. I., Grześkiewicz, A. M., Kubicki, M., Banti, C. N. & Hadjikakou, S. K. (2025). *Inorg. Chem. Commun.* **183**, 115915.

[bb3] Deswal, Y., Asija, S., Tufail, A., Dubey, A., Deswal, L., Kumar, N. & Barwa, P. (2024). *J. Inorg. Organomet. Polym. Mater.* **34**, 144–160.

[bb4] Dolomanov, O. V., Bourhis, L. J., Gildea, R. J., Howard, J. A. K. & Puschmann, H. (2009). *J. Appl. Cryst.* **42**, 339–341.

[bb5] El–Sebaey, S. A. (2020). *ChemistrySelect* **5**, 11654-11680.

[bb6] El-Sherief, H. A., Youssif, B. G., Bukhari, S. N. A., Abdel-Aziz, M. & Abdel-Rahman, H. M. (2018). *Bioorg. Chem.* **76**, 314–325. 10.1016/j.bioorg.2017.12.013 29227915

[bb7] Groom, C. R., Bruno, I. J., Lightfoot, M. P. & Ward, S. C. (2016). *Acta Cryst.* B**72**, 171–179. 10.1107/S2052520616003954 PMC4822653 27048719

[bb8] Gultekin, E., Kolcuoglu, Y., Akdemir, A., Sirin, Y., Bektas, H. & Bekircan, O. (2018). *ChemistrySelect* **3**, 8813-8818.

[bb9] Kaur, P. & Chawla, A. (2017). *Int. Res. J. Pharm.* **8**, 10–29.

[bb10] Khayrullaev, G., Torambetov, B., Kadirova, S. & Vaksler, Y. (2023). *Z. Kristallogr. New Cryst. Struct.* **238**, 141–144.

[bb11] Lin, S., Cui, Y. Z., Qiu, Q. M., Han, H. L., Li, Z. F., Liu, M., Xin, X. L. & Jin, Q. H. (2017). *Polyhedron* **134**, 319–329.

[bb12] Ma, C., Li, Y., Han, Y. & Zhang, R. (2008). *Inorg. Chim. Acta* **361**, 380–386.

[bb13] Naeem, N., Mughal, E. U., Sadiq, A., Othman, G. A. & Shakoor, B. (2025). *Arch. Pharm.* **358**, e70059. 10.1002/ardp.70059 40726245

[bb14] Nuralieva, G., Alieva, M., Torambetov, B., Leslee, D. B. C., Senthilkumar, B., Kaur, S., Dabke, N. B., Vanka, K., Ashurov, J., Kadirova, S. & Gonnade, R. G. (2025). *J. Mol. Struct.* **1338**, 142274.

[bb15] Pirimova, M., Torambetov, B., Kadirova, S., Ziyaev, A., Gonnade, R. G. & Ashurov, J. (2022). *Acta Cryst.* E**78**, 794–797. 10.1107/S2056989022006922 PMC9361377 35974814

[bb16] Rakova, O. A., Sanina, N. A., Aldoshin, S. M., Goncharova, N. V., Shilov, G. V., Shulga, Y. M. & Ovanesyan, N. S. (2003). *Inorg. Chem. Commun.* **6**, 145–148.

[bb17] Rigaku OD (2021). *CrysAlis PRO*. Rigaku Oxford Diffraction, Yarnton, England.

[bb18] Sathyanarayana, R. & Poojary, B. (2020). *J. Chin. Chem. Soc.* **67**, 459–477.

[bb19] Sheldrick, G. M. (2015*a*). *Acta Cryst.* A**71**, 3–8.

[bb20] Sheldrick, G. M. (2015*b*). *Acta Cryst.* C**71**, 3–8.

[bb21] Spackman, M. A. & Jayatilaka, D. (2009). *CrystEngComm* **11**, 19–32.

[bb22] Spackman, M. A. & McKinnon, J. J. (2002). *CrystEngComm* **4**, 378–392.

[bb23] Spackman, P. R., Turner, M. J., McKinnon, J. J., Wolff, S. K., Grimwood, D. J., Jayatilaka, D. & Spackman, M. A. (2021). *J. Appl. Cryst.* **54**, 1006–1011. 10.1107/S1600576721002910 PMC8202033 34188619

[bb24] Torambetov, B., Khojabaeva, G., Bharty, M. K., Gupta, S. K., Kadirova, S., Pradeep, S., Dastager, S. G. & Gonnade, R. G. (2025). *J. Mol. Struct.* **1354**, 144763.

[bb25] Wen, X., Zhou, Y., Zeng, J. & Liu, X. (2020). *Curr. Top. Med. Chem.* **20**, 1441–1460. 10.2174/1568026620666200128143230 31994462

[bb26] Yang, W., Qiu, Q.-M., Jin, Q.-H. & Zhang, C.-L. (2012). *Acta Cryst.* E**68**, o3194. 10.1107/S1600536812042742 PMC3515285 23284505

[bb27] Zhang, R. B., Li, Z. J., Cheng, J. K., Qin, Y. Y., Zhang, J. & Yao, Y. G. (2008). *Cryst. Growth Des.* **8**, 2562–2573.

